# Work-Related Musculoskeletal Disorders and Quality of Life in Nursing Personnel Across Northwestern Greece

**DOI:** 10.7759/cureus.86022

**Published:** 2025-06-14

**Authors:** Nikolaos Sontis, Anastasios Korompilias, Aimilios Pakos, Ioannis Gkiatas, Ioannis D Gelalis

**Affiliations:** 1 Department of Orthopaedic Surgery and Traumatology, University Hospital of Ioannina, Ioannina, GRC

**Keywords:** healthcare professionals, musculoskeletal, nursing, quality of life, risk factors

## Abstract

Introduction: Work-related musculoskeletal disorders (WrMSDs) among nursing personnel have been recognized as one of the most frequent musculoskeletal injuries. The aim of the present study was to estimate the incidence of WrMSDs in a sample of nursing personnel, identify predictors of WrMSDs, and examine their effects on quality of life (QoL).

Methodology: A cross-sectional design was adopted, and participants were full-time nursing personnel at five hospitals in northwestern Greece. Data included demographics, the RAND Short Form health survey (RAND SF-36) to measure the physical (Physical Component Score, PCS) and mental (Mental Component Score, MCS) components of QoL, and the Nordic Musculoskeletal Questionnaire (NMQ) to assess WrMSDs. Data were analyzed descriptively to summarize main outcome measures, and linear models were used to estimate the effect of WrMSDs on QoL.

Results: A total of 402 participants (n = 350 females) were included. WrMSDs were prevalent in 49.5% of participants (n = 199), with the lumbar spine being the most frequently affected site (56%). WrMSDs were most strongly associated with moving patients (p < 0.001), lifting or carrying objects from the ground (p < 0.001), and the degree or intensity of heavy object lifting (p = 0.002). Nursing personnel with a WrMSD diagnosis had an average PCS score that was 5.7 points lower (95% CI: -7.4 to -4.1; p < 0.001) than those without a WrMSD diagnosis. Participants with 20-24 and >25 years of working experience had PCS scores 5.9 (95% CI: -9.1 to -2.6; p < 0.001) and 6.6 (95% CI: -9.7 to -3.5; p < 0.001) points lower, respectively, than those with <5 years of experience. Nurses and nursing supervisors/assistant supervisors had PCS scores 3.1 (95% CI: 1.3 to 4.9; p < 0.001) and 4.1 (95% CI: 0.9 to 7.3; p < 0.001) points higher, respectively, than nursing assistants. Participants with a 20-minute break had an MCS score 4.3 points higher (95% CI: 0.4 to 8.2; p = 0.032) than those with no break. Participants from GHF hospital had MCS scores 5.8 points lower (95% CI: -9.2 to -2.3; p < 0.001) than those from the University Hospital of Ioannina (UHI).

Conclusion: Nearly half of the participants were diagnosed with WrMSDs, with the lumbar spine being the most commonly affected area. Several nursing tasks were significantly associated with the occurrence of WrMSDs. The physical component of QoL was negatively affected by the presence of WrMSDs, as well as by seniority and job position. In contrast, WrMSDs did not impact the mental component of QoL. Interventions aimed at reducing the incidence of WrMSDs may improve physical health in nursing personnel; however, improving mental well-being may require additional targeted strategies.

## Introduction

Work-related musculoskeletal disorders (WrMSDs) have been recognized as one of the most frequent musculoskeletal injuries [1,2], and nursing personnel are characterized as a high-risk group for developing WrMSDs [3-5]. In addition, WrMSDs are considered one of the leading causes of occupational diseases and are associated with the development of chronic pain among healthcare professionals [6].

The anatomical site with the highest prevalence of WrMSDs among registered nurses is the spine, more specifically the lumbar (~60%) and cervical (~53%) spine, as well as the shoulder level (47%) [3,7]. In clinical practice, nursing personnel are frequently required to perform tasks that involve excessive flexion and rotation, such as moving and/or lifting objects above shoulder level and handling patients by moving, pulling, or pushing them to and from beds or other locations [8,9]. The reported high frequency of WrMSDs among nursing personnel can lead to workforce reductions, an increased number of sick days per year, and professional burnout [10-12]. In addition, WrMSDs negatively affect life outside the workplace by deteriorating quality of life (QoL) [13,14], although others report no impact [15].

The nature of WrMSDs is multifactorial. Obesity, age, years of employment, work tasks, number of weekly working hours, excessive night shifts, stress levels, job satisfaction, and exercise habits are considered risk factors for developing WrMSDs [2]. Unraveling the incidence of WrMSDs and its relationship with underlying factors among nursing personnel is a key step for health policy administrators to design and implement effective interventions for healthcare professionals [16]. Therefore, the aims of the present study were to document the incidence of WrMSDs in a sample of nursing personnel at five hospitals in a large region in northwestern Greece, identify demographic and other predictors, and examine their effect on QoL. We hypothesized that the incidence would be high (40-60%) and similar to previously reported data, and that diagnosis would be associated with reduced QoL.

## Materials and methods

The study was approved by the Institutional Review Board of the University Hospital of Ioannina (UHI). Participants were fully informed of the voluntary nature of study participation. All data were anonymous and could be accessed only by the research team.

Nursing staff working in public hospitals of Epirus, namely, UHI, General Hospital of Ioannina "G. Hatzikosta" (GHI), General Hospital of Preveza (GHP), General Hospital of Arta (GHA), and General Hospital of Filiaton Thesprotia (GHF), were included in the study. Inclusion criteria for the purposes of the present study were a valid license to practice nursing and current employment at one of the sampled hospitals. Exclusion criteria were pregnancy, chronic conditions that may affect the musculoskeletal system (e.g., autoimmune diseases, arthritis), and recent (within the last six months) major injury that affected the musculoskeletal system (e.g., ruptured meniscus).

These hospitals were selected because they receive a large number of patients daily and employ a competent sample of nursing staff of varying educational levels and a wide range of professional experience.

A total of 500 questionnaires were distributed to randomly selected members of the nursing staff across a wide range of departments and clinics (departments of pathology, surgery, pediatrics, emergency departments, and intensive care units), as well as to staff of different educational levels. Questionnaires were distributed during the morning shift due to the higher number of employees working during that period. Of the 500 questionnaires distributed, 402 were returned completed, resulting in an overall response rate of 80.4%.

Data collection

Data collection took place from January 2024 to December 2024, using a structured questionnaire comprising three sections (Appendices). The first section gathered sociodemographic information, including personal and anatomical characteristics, work sector, hospital workplace, years of experience in the current position, and workplace environment.

The second section incorporated the Nordic Musculoskeletal Questionnaire (NMQ), developed by Kuorinka et al. [17], a validated self-administered screening tool designed to assess the prevalence of musculoskeletal disorders within a population. In the present study, the NMQ was used to analyze the relationship between the workplace environment and WrMSDs. The questionnaire has been translated, culturally adapted, and validated for use in Greek [18]. While it has been previously administered to residents of rural Crete [19], it has not yet been used among nursing staff in Epirus. The NMQ is freely available for research and educational use; thus, no specific permission for non-commercial application was obtained.

The third section included the RAND Short Form-36 version 1, which is a publicly available tool for non-commercial use and does not require licensing or permission. The instrument and scoring instructions are freely distributed by the RAND Corporation and are widely used in academic research to assess the overall QoL [20]. The RAND SF-36 consists of 36 Likert-type items evaluating participants’ ability to perform daily activities. These 36 items are grouped into eight underlying dimensions (bodily function, physical role, emotional role, mental health, physical pain, general health, vitality, and social functioning). Responses are scored on a scale from 0 to 100, with higher scores indicating better QoL. Two-component summary scores differentiate between physical health (Physical Component Score, PCS) and mental health (Mental Component Score, MCS). The RAND SF-36 has demonstrated reliability and validity in previous research [21].

Statistical analysis

Data were originally collected using a custom-made Excel spreadsheet form (Microsoft Corporation, Redmond, Washington). All statistical analyses were performed within the environment of the open programming language R (version 4.2.1, R Foundation for Statistical Computing, Vienna, Austria). Study outcomes were the diagnosis of WrMSDs (by a certified MD) within the last six months, the PCS score regarding physical QoL, and the MCS score regarding mental QoL. Continuous variables are presented as mean ± SD, whereas categorical variables are presented as frequencies and percentages. Chi-square tests were used to evaluate the association between the incidence of WrMSD medical diagnosis and categorical predictors. The Pearson correlation coefficient was used to examine the association between PCS score and MCS score. The magnitude of the correlation was evaluated using the thresholds proposed by Cohen [22]: trivial <0.1, small (0.11-0.30), moderate (0.31-0.50), large (0.51-0.70), very large (0.71-0.90), and almost perfect (>0.90).

General linear models were used to estimate the effect of WrMSD medical diagnosis on QoL after adjustment for demographic and workplace characteristics. A “top-down” approach was used, where all possible predictors were initially considered, and variables that did not reach statistical significance were removed in an iterative procedure [23]. The final model reported was chosen based on model parsimony and interpretability. For all analyses, the level of statistical significance was set at p = 0.05.

## Results

There were a total of 402 participants, and the majority were female (87.1%, n = 350). Approximately 90% were currently employed as a nurse or nursing assistant, and around 42% were senior staff with more than 25 years of working experience. The incidence of WrMSD in our study sample was 49.5% (n = 199), as shown in Table 1.

**Table 1:** Demographic and clinical characteristics of study participants (n=402) BMI: body mass index, PCS: Physical Component Score, MCS: Mental Component Score, WrMSD: work-related musculoskeletal disorder.

Variable	Values
Age, years	47.2 (±8.0)
Weight, kg	71.5 (±12.5)
Height, m	1.67 (±0.07)
BMI, m·kg^-2^	25.7 (±3.9)
PCS	43.5 (±9.2)
MCS	43.1 (±9.8)
Gender	
Male	52 (12.9%)
Female	350 (87.1%)
Current position	
Nursing supervisor/assistant supervisor	33 (8.2%)
Nurse	218 (54.2%)
Nursing assistant	143 (35.6%)
Midwife	8 (2.0%)
Body type	
Lean	4 (1.0%)
Normal	268 (66.7%)
Overweight	130 (32.3%)
Nursing seniority, years	
<5	36 (9.0%)
5-9	24 (6.0%)
10-14	35 (8.7%)
15-20	58 (14.4%)
20-24	81 (20.1%)
>25	168 (41.8%)
Diagnosis of WrMSD	
No	203 (50.5%)
Yes	199 (49.5%)

The most frequent anatomical site for developing musculoskeletal complaints is the lumbar spine (55.5%), followed by the head and cervical spine (34.8%), shoulder (24.1%), wrist (23.9%), and knee joint (22.1%) (Table 2).

**Table 2:** Frequency of reported musculoskeletal complaints per anatomical site

Anatomical site	n	%
Head and cervical spine	140	34.8%
Shoulder	97	24.1%
Humerus	19	4.7%
Elbow	37	9.2%
Forearm	22	5.5%
Wrist	96	23.9%
Hand	43	10.7%
Lumbar spine	223	55.5%
Hip	50	12.4%
Knee	89	22.1%
Gastrocnemius	20	5.0%
Ankle joint	52	12.9%
Foot	26	6.5%

The association of potential demographic predictors of the WrMSD diagnosis is presented in Table 3. The incidence of WrMSD diagnosis had a strong association with the factor hospital as well as with nursing seniority.

**Table 3:** Association between WrMSD diagnosis and demographic characteristics p-value = level of significance for the corresponding chi-square test. All p-values <0.05 are considered statistically significant. Values are given as n (%) per factor level. UHI: University Hospital of Ioannina, GHI: General Hospital of Ioannina "G. Hatzikosta", GHP: General Hospital of Preveza, GHA: General Hospital of Arta, GHF: General Hospital of Filiaton Thesprotia, BMI: body mass index, WrMSD: work-related musculoskeletal disorders, χ2: chi-squared, df: degrees of freedom.

Variables	WrMSD (no, n=203)	WrMSD (yes, n=199)	Statistics	df	p-value
Hospital					
UHI	83 (40.1%)	124 (59.9%)	χ^2^=18.714	4	<0.001
GHI	27 (58.7%)	19 (41.3%)			
GHP	19 (61.3%)	12 (38.7%)			
GHA	50 (63.3%)	29 (36.7%)			
GHF	24 (61.5%)	15 (38.5%)			
Gender					
Male	31 (59.6%)	21 (40.4%)	χ^2^=1.986	1	0.159
Female	172 (49.1%)	178 (50.9%)			
Current position					
Nursing supervisor/assistant supervisor	14 (42.4%)	19 (57.6%)	χ^2^=2.235	3	0.525
Nurse	117 (53.7%)	110 (46.3%)			
Assistant nurse	68 (47.6%)	75 (52.4%)			
Midwife	4 (50.0%)	4 (50.0%)			
Nursing seniority					
<5 years	29 (80.6%)	7 (19.4%)	χ^2^=22.87	5	<0.001
5-9 years	14 (58.3%)	10 (41.7%)			
10-14 years	22 (62.9%)	13 (37.1%)			
15-19 years	31 (53.4%)	27 (46.6%)			
20-24 years	39 (48.1%)	42 (51.9%)			
25+ years	68 (40.5%)	100 (59.5%)			
BMI classification					
Normal	98 (55.4%)	79 (44.6%)	χ^2^=3.106	2	0.212
Overweight	79 (47.3%)	88 (52.7%)			
Obese	26 (44.8%)	32 (55.2%)			

The incidence of WrMSD diagnosis had a strong association with several nursing tasks, including moving patients, transporting materials with and without a wheelbarrow, prolonged sedentary activity, lifting or carrying objects, and lifting or carrying patients (Table 4).

**Table 4:** Association between WrMSD diagnosis and nursing tasks p-value = level of significance for the corresponding chi-square test. All p-values <0.05 are considered statistically significant. Values are given as n (%) per factor level. WrMSD: work-related musculoskeletal disorder, χ2: chi-squared, df: degrees of freedom.

Variable	WrMSD (no, n=203)	WrMSD (yes, n=199)	Statistics	df	p-value
Patient transport via stretchers					
No	137 (49.8%)	138 (50.2%)	χ^2^=0.161	1	0.689
Yes	66 (52.0%)	61 (48.0%)			
Moving patients					
No	92 (59.4%)	63 (40.6%)	χ^2^=7.917	1	0.005
Yes	111 (44.9%)	136 (55.1%)			
Transporting material with a wheelbarrow					
No	61 (62.2%)	37 (37.8%)	χ^2^=7.154	1	0.007
Yes	142 (46.7%)	162 (53.3%)			
Transporting material w/o a wheelbarrow					
No	69 (61.1%)	44 (38.9%)	χ^2^=7.018	1	0.008
Yes	134 (46.4%)	155 (53.6%)			
Sorting material on high shelves					
No	23 (65.7%)	12 (34.3%)	χ^2^=3.551	1	0.060
Yes	180 (49.0%)	187 (51.0%)			
Sorting material on low shelves					
No	23 (62.2%)	14 (37.8%)	χ^2^=2.218	1	0.136
Yes	180 (49.3%)	185 (50.7%)			
Changing patient clothes					
No	73 (56.6%)	56 (43.4%)	χ^2^=2.820	1	0.093
Yes	130 (47.3%)	143 (52.4%)			
Bathing patients					
No	114 (52.5%)	103 (47.5%)	χ^2^=0.783	1	0.376
Yes	89 (48.1%)	96 (51.9%)			
Sedentary work (i.e., prolonged sitting)					
No	79 (59.4%)	54 (40.6%)	χ^2^=6.299	1	0.012
Yes	124 (46.1%)	145 (53.9%)			
Preparing patient regimes					
No	57 (50.4%)	56 (49.6%)	χ^2^=<0.001	1	0.989
Yes	146 (50.5%)	143 (49.5%)			
Executing patient regimes					
No	55 (50.0%)	55 (50.0%)	χ^2^=0.015	1	0.903
Yes	148 (50.7%)	144 (49.3%)			
Lifting/carrying objects from the ground					
No	72 (64.9%)	39 (35.1%)	χ^2^=12.662	1	<0.001
Yes	131 (45.0%)	160 (55.0%)			
Lifting/carrying patients					
No	78 (59.5%)	53 (40.5%)	χ^2^=6.359	1	0.012
Yes	125 (46.1%)	146 (53.9%)			

The incidence of WrMSD diagnosis had no association with most workplace characteristics; the only strong association was observed between the incidence of WrMSD diagnosis and the degree or intensity of heavy object lifting during nursing tasks (Table 5).

**Table 5:** Association between WrMSD diagnosis and work place characteristics p-value = level of significance for the corresponding test. All p-values <0.05 are considered statistically significant. Values are given as n (%) per factor level. WrMSD: work-related musculoskeletal disorder, χ2: chi-squared, df: degrees of freedom, FE: Fisher’s exact test.

Variable	WrMSD (no, n=203)	WrMSD (yes, n=199)	Statistics	df	p-value
Workplace spacing					
Spacious	9 (60.0%)	6 (40.0%)	χ^2^=0.763	3	0.858
Adequate	70 (50.7%)	68 (49.3%)			
Limited	96 (50.5%)	94 (49.5%)			
Inadequate	28 (47.5%)	31 (52.5%)			
Workplace distances					
Short	37 (62.7%)	22 (37.3%)	χ^2^=4.219	2	0.121
Average	116 (48.9%)	121 (51.1%)			
Long	50 (47.2%)	56 (52.8%)			
Degree/intensity of prolonged standing					
High	138 46.9%)	156 (53.1%)	χ^2^=5.563	2	0.062
Average	58 (60.4%)	38 (39.6%)			
Low	7 (58.3%)	5 (41.7%)			
Degree/intensity of prolonged walking					
High	81 (51.6%)	76 (48.4%)	χ^2^=1.930	2	0.381
Average	68 (54.0%)	58 (46.0%)			
Low	54 (45.4%)	65 (54.6%)			
Prolonged inappropriate body position					
High	83 (48.0%)	90 (52.0%)	χ^2^=3.536	2	0.171
Average	71 (48.3%)	76 (51.7%)			
Low	49 (59.8%)	33 (40.2%)			
Prolonged awkward body position					
High	50 (41.3%)	71 (58.7%)	χ^2^=5.856	2	0.053
Average	78 (54.9%)	64 (45.1%)			
Low	75 (54.0%)	64 (46.0%)			
Prolonged bending					
High	54 (45.4%)	65 (54.6%)	χ^2^=1.994	2	0.369
Average	77 (51.3%)	73 (48.7%)			
Low	72 (54.1%)	61 (45.9%)			
Prolonged hyperextension					
High	35 (39.3%)	54 (60.7%)	χ^2^=5.721	2	0.053
Average	66 (54.1%)	56 (49.5%)			
Low	102 (53.4%)	89 (46.6%)			
Degree/intensity of heavy object lifting					
High	38 (35.8%)	68 (64.2%)	χ^2^=12.374	2	0.002
Average	68 (56.2%)	53 (43.8%)			
Low	97 (55.4%)	78 (44.6%)			
Degree/intensity of moving heavy objects					
High	46 (41.8%)	64 (58.2%)	χ^2^=4.985	2	0.083
Average	64 (56.1%)	50 (43.9%)			
Low	93 (52.2%)	85 (47.8%)			
Degree/intensity of moving patients from the bed					
High	66 (42.9%)	88 (57.1%)	χ^2^=5.895	2	0.052
Average	59 (56.2%)	46 (43.8%)			
Low	78 (54.5%)	65 (45.5%)			
Degree/intensity of moving patients from/onto the stretcher					
High	48 (42.5%)	65 (57.5%)	χ^2^=6.160	2	0.046
Average	65 (59.1%)	45 (40.9%)			
Low	90 (50.3%)	89 (49.7%)			
Degree/intensity of prolonged sitting					
High	11 (40.7%)	16 (59.3%)	χ^2^=1.442	2	0.486
Average	62 (53.4%)	54 (46.6%)			
Low	130 (50.2%)	129 (49.8%)			
Duration of break					
No break	37 (59.5%)	47 (40.5%)	FE=10.141	5	0.071
v≤5 min	28 (48.3%)	30 (51.7%)			
5-10 min	73 (56.2%)	57 (43.8%)			
10-15 min	40 (47.1%)	45 (52.9%)			
15-20 min	16 (45.7%)	19 (54.3%)			
20-30 min	9 (90.0%)	1 (10.0%)			

**Figure 1:**
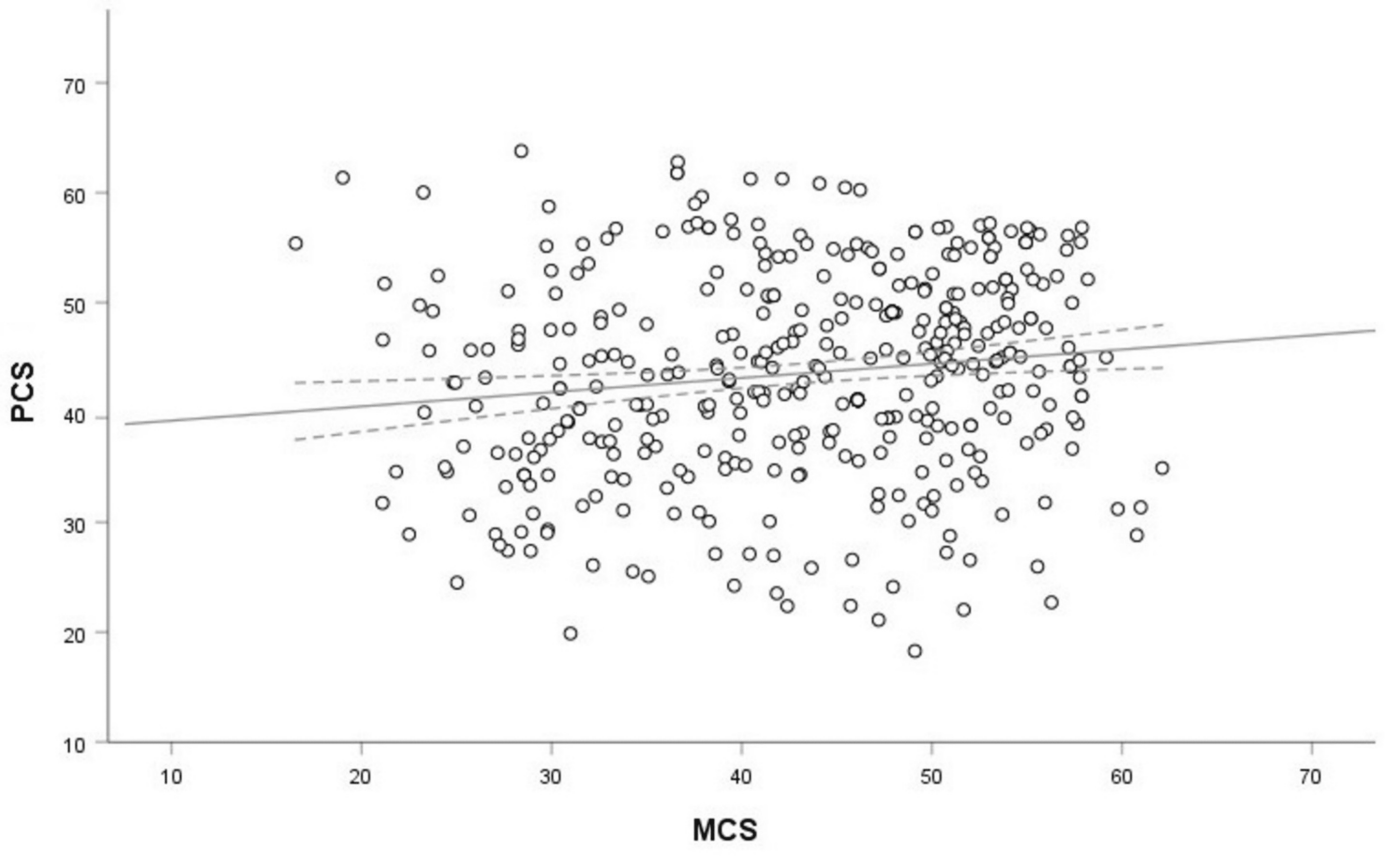
Scatterplot showing the association between PCS and MCS Open circles indicate the corresponding PCS and MCS raw data for each participant. Black solid line indicates regression line with 95% confidence intervals (dotted lines). PCS: Physical Component Score, MCS: Mental Component Score.

There was a statistically significant, positive but small correlation between the physical (PCS) and mental (MCS) dimensions of QoL (r = 0.14, 95% CI (0.04, 0.24), p = 0.005). Thus, PCS and MCS are largely unrelated variables (Figure 1).

WrMSD diagnosis, nursing seniority, and position were statistically significant predictors of PCS (Table 6). On the contrary, hospital, gender, educational background, BMI classification, and break duration had no impact on PCS. More specifically, nursing personnel with a WrMSD diagnosis had −5.7 (−7.4; −4.1) lower PCS on average compared to nursing personnel without a WrMSD diagnosis (p < 0.001). Participants with 20-24 and >25 years of working experience had −5.9 (−9.1; −2.6) and −6.6 (−9.7; −3.5), respectively, lower PCS on average compared to nursing personnel with <5 years of working experience (p < 0.001). Finally, nurse and nursing supervisors/assistant supervisors had 3.1 (1.3; 4.9) and 4.1 (0.9; 7.3) respectively higher PCS on average compared to nursing assistants (p < 0.001).

**Table 6:** Predictors of the physical component of QoL in nursing personnel. Estimates are presented as mean % (95%CI). WrMSD: work-related musculoskeletal disorder, RSE: residual standard error, QoL: quality of life.

Effect	Estimate	p-value
Intercept	49.0 (46.0; 52.0)	<0.001
WrMSD diagnosis_yes_	-5.7 (-7.4; -4.1)	<0.001
Working experience_5-9years_	-1.3 (-5.6; 3.0)	0.559
Working experience_10-14years_	-2.5 (-6.3; 1.4)	0.209
Working experience_15-19years_	-3.2 (-6.6; 0.3)	0.075
Working experience_20-24years_	-5.9 (-9.1; -2.6)	<0.001
Working experience_>25years_	-6.6 (-9.7; -3.5)	<0.001
Position_midwife_	2.5 (-3.5; 8.5)	0.417
Position_nurse_	3.1 (1.3; 4.9)	<0.001
Position_supervisor/ass. supervisor_	4.1 (0.9; 7.3)	0.012
F	12.57	<0.001
RSE	8.2	n/a
R^2^	20.6	n/a

Predictors of the mental dimension of QoL (MCS) are presented in Table 7. Hospital and break duration were statistically significant predictors of MCS. On the contrary, WrMSD diagnosis, nursing seniority, position, gender, educational background, and BMI classification had no impact on MCS. Participants with a break duration of 20 min had 4.3 (0.4; 8.2) higher MCS on average compared to nursing personnel with no break (p = 0.032). Finally, participants from GHF had −5.8 (−9.2; −2.3) lower MCS on average compared to participants from UHI (p < 0.001).

**Table 7:** Predictors of the mental component of QoL in nursing personnel. Estimates are presented as mean % (95%CI). RSE: residual standard error, QoL: quality of life.

Effect	Estimate	p-value
Intercept	43.2 (41.0; 45.4)	<0.001
Break duration_5min_	1.3 (-2.0; 4.7)	0.436
Break duration_10min_	0.6 (-2.1; 3.3)	0.674
Break duration_15min_	1.3 (-1.7; 4.2)	0.389
Break duration_20min_	4.3 (0.4; 8.2)	0.032
Break duration_30min_	3.1 (-3.4; 9.5)	0.351
Hospital_GHI_	-2.1 (-5.2; 1.1)	0.193
Hospital_GHA_	-2.0 (-4.6; 0.6)	0.133
Hospital_GHP_	-0.7 [-4.4; 3.0]	0.716
Hospital_GHF_	-5.8 (-9.2; -2.3)	<0.001
F	3.189	0.038
RSE	9.7	n/a
R^2^	4.0	n/a

## Discussion

The presented study documented the incidence of WrMSDs in a sample of nursing personnel across five hospitals in a large region in Northwestern Greece, identified demographic and other predictors of WrMSDs, and examined the effect of WrMSDs on QoL. Our research hypothesis was that the incidence of WrMSDs would be high (40-60%) and similar to previously reported data, and that the diagnosis of WrMSDs would be associated with reduced QoL.

The incidence of WrMSD in our study sample was 49.5% (n = 199). Previous studies suggest that the prevalence of WMSDs among nurses can range from 30% to over 70%, depending on specific job roles, hospital settings, and geographical locations [2-5]; thus, our results lie within approximately the median incidence reported in the literature. For example, on the high end of the incidence rate, Tinubu et al. [24] reported a prevalence of 78% among Nigerian nurses, while Yan et al. [25] observed a prevalence of 77.4% among Chinese nurses. On the low end of the incidence rate, Clari et al. [4], in a multicenter cross-sectional study, showed that 48.3% of operating room nurses reported one or more episodes of upper limb pain.

Furthermore, our results indicated that the most frequent anatomical site for developing musculoskeletal complaints was the lumbar spine (55.5%), followed by the head and cervical spine (34.8%), shoulder (24.1%), wrist (23.9%), and knee joint (22.1%). Our data are in line with previous reports [2,3,8,15,26,27]. Most of these studies employed the Nordic Musculoskeletal Questionnaire, which further enhances the validity of our results.

We observed that working seniority was the demographic variable with the strongest association with WrMSD diagnosis. In addition, the incidence of WrMSD diagnosis had a strong association with several nursing tasks (moving patients, transporting materials with and without wheelbarrows, prolonged sedentary activity, lifting/carrying objects, and lifting/carrying patients); however, we did not observe any association with most of the workplace characteristics. The only strong association was between the incidence of WrMSD diagnosis and the degree or intensity of heavy object lifting nursing tasks. These results are supported by previous meta-analytical findings indicating that the physical demands of the nursing profession (i.e., bending and twisting), as well as the physical demands of nurse-patient interaction (i.e., turning, dressing, bathing, seating, transferring patients), are considered risk factors for the development of WrMSD [2,7].

WrMSD diagnosis, nursing seniority, and position were statistically significant predictors of PCS. Previous studies report mixed findings on the effect of WrMSD on QoL [13-15], while the effect of seniority has not been examined thoroughly [24]. In addition, both nursing personnel and nursing supervisors or assistant supervisors reported significantly better QoL compared to assistant nurses, although current position was not associated with WrMSD diagnosis. These results may further indicate the unique effects of the above variables on QoL and that factors unrelated to WrMSD are responsible for the lower QoL in nursing assistants and more senior personnel. Regarding MCS, the only statistically significant predictors were hospital and break duration, while WrMSD had no impact on MCS. It appears that a sufficient amount of break time during the nursing shift is necessary for alleviating work-related stress [13-15]. It is worth noting that the participants of the present study reported PCS and MCS scores that are considered relatively low compared to the adult population worldwide [28].

The main limitation of the present study is that data regarding the occurrence of WrMSD were based on self-reported information provided by the study participants, and no actual objective testing was conducted to confirm the diagnosis. Thus, WrMSD caused by non-work-related factors cannot be eliminated. In addition, the study may be subject to selection bias, as individuals experiencing musculoskeletal symptoms, particularly those perceived to be work-related, might have been more motivated to participate. Distributing the questionnaire primarily during morning shifts may have excluded night shift workers or those with irregular schedules, who may differ in exposure or risk. These factors may limit the generalizability of the findings. A further limitation is that the Nordic Musculoskeletal Questionnaire does not assess comorbidities or coexisting health conditions, which may influence the reported symptoms and act as potential confounders. Future research should consider integrating broader health assessment tools to better account for these factors. Finally, while we investigated five hospitals in Northwestern Greece, approximately 50% of the participants were employed in the largest hospital of the area (UHI). While this may introduce some bias in the reported results, it also increases the ecological validity of the results in the sense that most hospitals in smaller cities around Greece are considered understaffed in nursing and medical personnel.

## Conclusions

In conclusion, almost half of the participants had been diagnosed with WrMSD, with the lumbar spine being the most frequently affected site. Several nursing tasks were significantly associated with the occurrence of WrMSD. The physical component of QoL was negatively affected by the presence of WrMSD, as well as by nursing seniority and job position. On the contrary, WrMSD had no impact on the mental component of QoL. These findings suggest that interventions aimed at reducing the incidence of WrMSDs may contribute to improvements in PCS among nursing personnel. However, targeted strategies beyond WrMSD prevention are necessary to enhance MCS in this population.

## Figures and Tables

**Figure 2:**
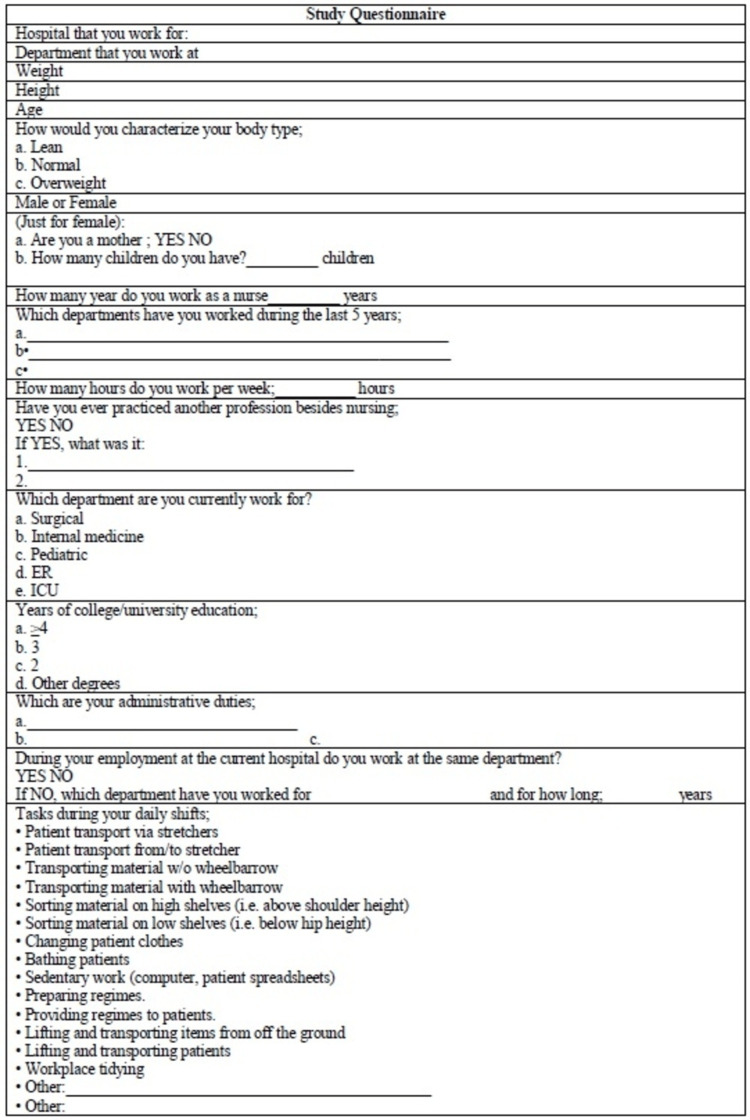
Study questionnaire (Part 1)

**Figure 3:**
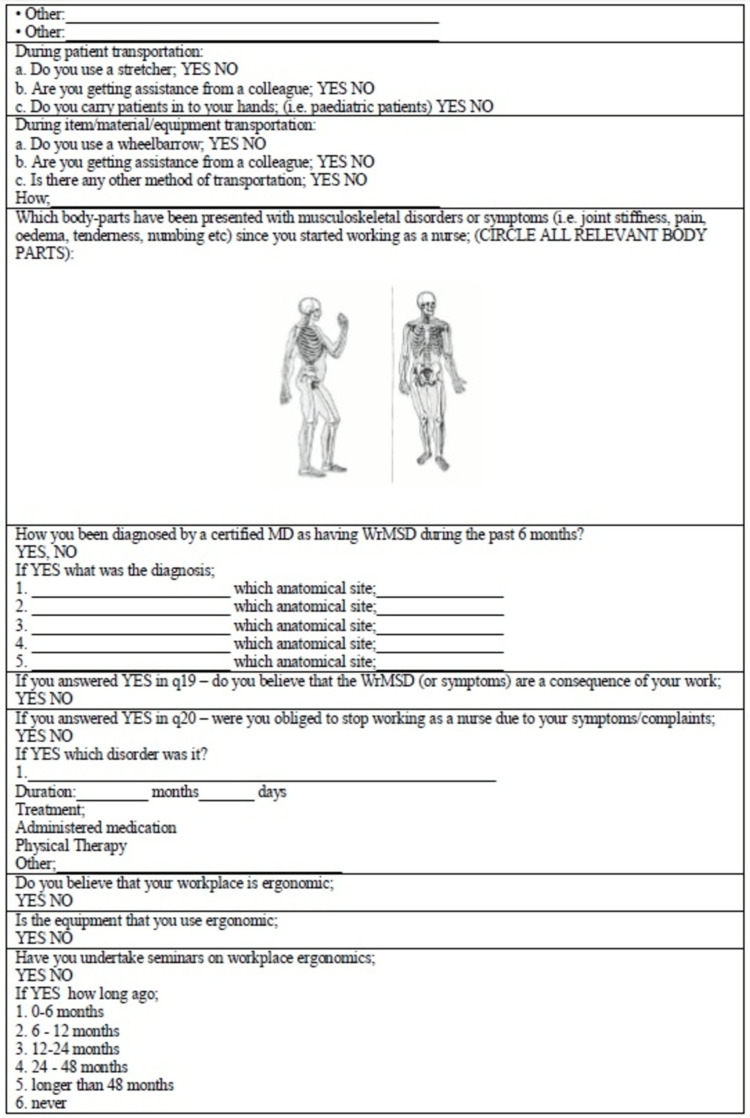
Study questionnaire (Part 2)

**Figure 4:**
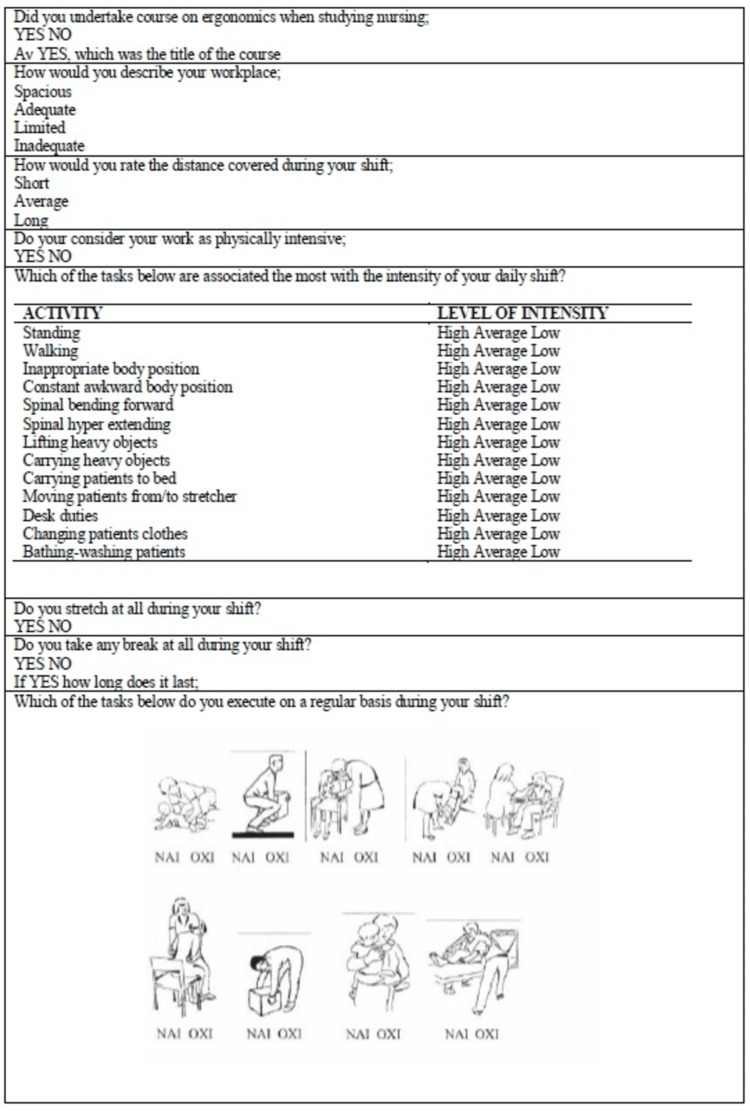
Study questionnaire (Part 3)

**Figure 5:**
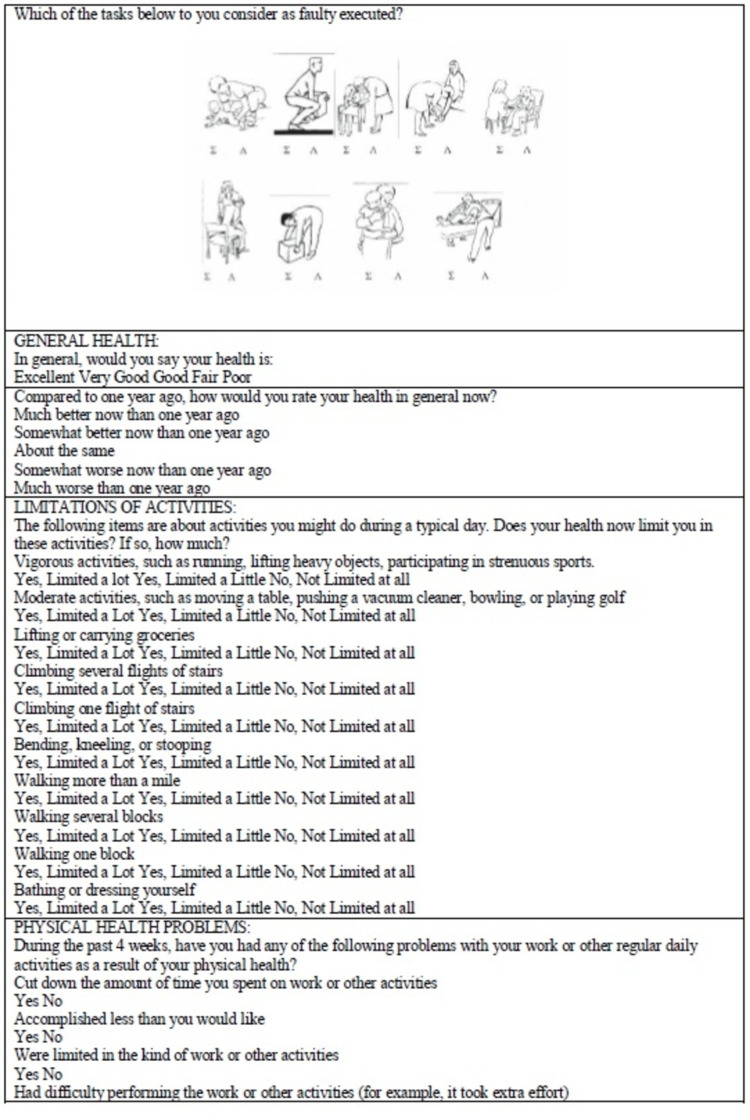
Study questionnaire (Part 4)

**Figure 6:**
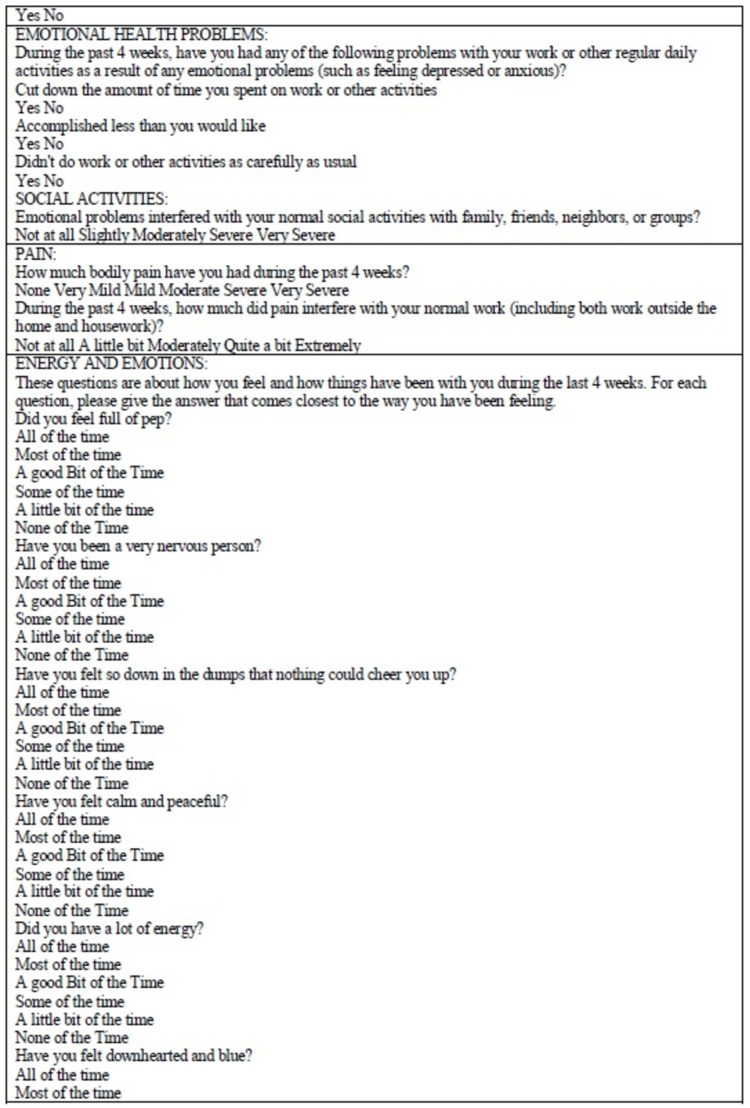
Study questionnaire (Part 5)

**Figure 7:**
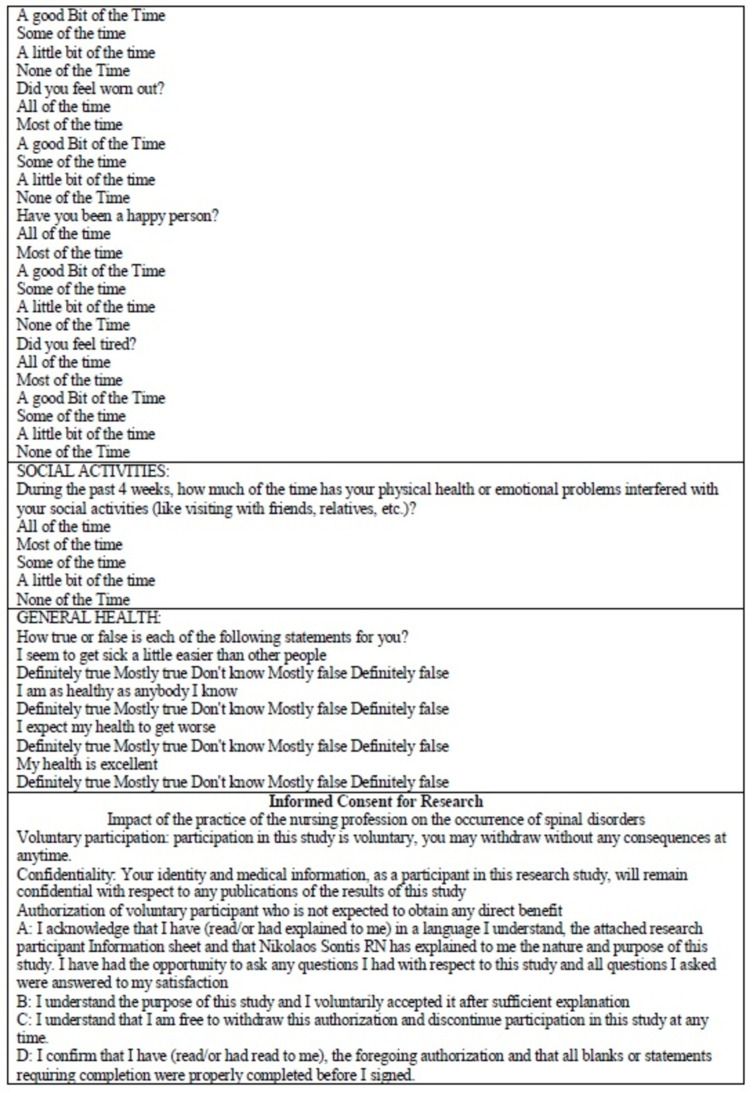
Study questionnaire (Part 6)
